# COVID-19 Pandemic and Pediatric Type 1 Diabetes: No Significant Change in Glycemic Control During The Pandemic Lockdown of 2020

**DOI:** 10.3389/fendo.2021.703905

**Published:** 2021-08-10

**Authors:** Benjamin Udoka Nwosu, Layana Al-Halbouni, Sadichchha Parajuli, Gabrielle Jasmin, Emily Zitek-Morrison, Bruce A. Barton

**Affiliations:** ^1^Division of Endocrinology, Department of Pediatrics, University of Massachusetts Medical School, Worcester, MA, United States; ^2^Department of Population and Quantitative Health Sciences, University of Massachusetts Medical School, Worcester, MA, United States

**Keywords:** COVID-19 pandemic, children, adolescents, type 1 diabetes, hemoglobin A1c

## Abstract

**Importance:**

There is no consensus on the impact of the 2020 COVID-19 pandemic lockdown on glycemic control in children and adolescents with type 1 diabetes (T1D) in the US.

**Aim:**

To determine the impact of the pandemic lockdown of March 15th through July 6^th^, 2020 on glycemic control after controlling for confounders.

**Subjects and Methods:**

An observational study of 110 subjects of mean age 14.8 ± 4.9 years(y), [male 15.4 ± 4.0y, (n=57); female 14.1 ± 3.8y, (n=53), p=0.07] with T1D of 6.31 ± 4.3y (95% CI 1.0-19.7y). Data were collected at 1-4 months before the lockdown and 1-4 months following the lifting of the lockdown at their first post-lockdown clinic visit.

**Results:**

There was no significant change in A1c between the pre- and post-pandemic lockdown periods, 0.18 ± 1.2%, (95% CI -0.05 to 0.41), p=0.13. There were equally no significant differences in A1c between the male and female subjects, -0.16 ± 1.2 *vs* -0.19 ± 1.2%, p=0.8; insulin pump users and non-pump users, -0.25 ± 1.0 *vs* -0.12 ± 1.4%, p=0.5; and pubertal *vs* prepubertal subjects, 0.18 ± 1.3 *vs* -0.11 ± 0.3%, p=0.6. The significant predictors of decrease in A1c were pre-lockdown A1c (p<0.0001) and the use of CGM (p=0.019). The CGM users had significant reductions in point-of-care A1c (0.4 ± 0.6%, p=0.0012), the CGM-estimated A1c (p=0.0076), mean glucose concentration (p=0.022), a significant increase in sensor usage (p=0.012), with no change in total daily dose of insulin (TDDI). The non-CGM users had significantly increased TDDI (p<0.0001) but no change in HbA1c, 0.06 ± 1.8%, p=0.86.

**Conclusions:**

There was no change in glycemic control during the pandemic lockdown of 2020 in US children.

## Introduction

The emergence of the global COVID-19 pandemic in 2019 triggered strict lockdown measures in various parts of the world to prevent the spread of the causative agent, the severe acute respiratory syndrome coronavirus-2 (SARS-CoV-2), leading to the restriction of movement and quarantining of individuals who either had the infection or were at risk for the disease. These measures severely interrupted the delivery of health care services to individuals with acute and chronic diseases such as type 1 diabetes (T1D). With the lifting of the initial lockdown measures, it has become necessary to assess the medium to long-term impact of the pandemic lockdown on glycemic control in children and adolescents with type 1 diabetes as a way of providing a historical context to the present pandemic and generating information to address diabetes care in future occurrences.

A review of current literature in endocrinology shows a lack of consensus on the impact of COVID-19 pandemic lockdown on glycemic control in patients with type 1 diabetes (1) as some studies reported either no change in glycemic control (2–4), an improvement in glycemic control (5–8), worsening glycemic control (9), or contradictory results in children, adolescents, and adults. There is a dearth of data from published studies that compared the changes in continuous glucose monitoring (CGM) metrics and the principal long-term glycemic marker, hemoglobin A1c (10), in children and adolescents with T1D during the lockdown.

The COVID-19 pandemic timeline in Massachusetts (11) started on February 1st, 2020 with the confirmation of the first patient with the disease in the state. This was followed with the closure of schools in Massachusetts on March 15^th^, 2020, as part of the strict stay-at-home period that lasted through May 18^th^, 2020. This was then followed by a gradual, phased reopening plan that began on June 2^nd^, 2020 and extended to July 6^th^, 2020. During this period, interactions between children and adolescents with type 1 diabetes and their caregivers occurred mostly through telehealth services and phone calls by nurses and physicians.

The aim of this study was to determine the impact of the pandemic lockdown of March 15th through July 6^th^, 2020 on glycemic control after controlling for confounders, by specifically investigating the differences in the long-term glycemic marker, A1c (10), and short-term markers, CGM metrics between the periods before and after the pandemic lockdown of 2020. Our hypothesis was that the pandemic lockdown would worsen glycemic control and result in significant elevations in A1c and a worsening of the CGM metrics in the post-lockdown period compared to the pre-lockdown months. Data on lipid parameters, 25-hydroxyvitamin D, and any record of infection with SARS-CoV-2 were collected as covariates.

## Subjects and Methods

### Ethics Statement

This study protocol was approved by the Institutional Review Board of the University of Massachusetts, under docket # H00015476. All subjects’ records were anonymized and de-identified prior to analysis.

### Subjects

The patient population consisted of 110 pediatric patients with a confirmed diagnosis of type 1 diabetes from the Children’s Medical Center Database of the UMassMemorial Medical Center, Worcester, Massachusetts, USA. The patient catchment area included the states of Massachusetts, Rhode Island, and New Hampshire. The pediatric endocrinology clinic treats approximately 550 patients with T1D, of whom about 45% (247) use the CGM either as a stand-alone device or in combination with an insulin pump. Of these, about 90 subjects are on stand-alone CGM device. All subjects had type 1 diabetes for > 1 year to exclude the effect of partial clinical remission on changes in A1c. Each subject had both the pre-pandemic and post-pandemic A1c data and thus served as his or her own control. Subjects were not randomly selected for inclusion in the study. Rather, subjects were included based on the availability of pre- and post-pandemic data on glycemic control. The diagnosis of type 1 diabetes (10) was established by any of the following glycemic parameters: a fasting blood glucose of ≥ 126 mg/dL (7 mmol/L), and/or 2-hour postprandial glucose of ≥200 mg/dL (11.1 mmol/L), and/or random blood glucose of ≥200 mg/dL with symptoms of polyuria and/or polydipsia, in addition to the detection of one or more diabetes-associated auto-antibodies, namely insulinoma-associated-2 autoantibodies, islet cell cytoplasmic autoantibodies, glutamic acid decarboxylase antibodies, and insulin autoantibodies. Patients with other forms of diabetes mellitus were excluded from the study. Retrospective data collection for anthropometric, clinical, and biochemical parameters were conducted as follows: pre-pandemic lockdown data were obtained from Nov 1^st^, 2019 through February 29^th^, 2020, and post-pandemic lockdown data were obtained from July 15th through November 31^st^, 2020. Specifically, data were collected at 1-4 months before the lockdown and 1-4 months following the lockdown at their first post-lockdown in-person clinic visit.

### Anthropometry

As described in detail previously (12), weight was measured to the nearest 0.1 kg using an upright scale. Height was measured to the nearest 0.1 cm using a wall-mounted stadiometer that was calibrated daily. BMI was calculated from the formula: weight/height^2^ (kg/m^2^), and expressed as z-score for age and sex, based on National Center for Health Statistics (NCHS) data (13). Overweight was defined as BMI of ≥85^th^ but <95^th^ percentile, and obesity was defined as BMI of ≥95^th^ percentile for age and gender. Sexual maturity rating was denoted by Tanner staging with puberty marked by Tanner II-V stages.

### Assays

The methodologies of assays for laboratory chemistries have been previously described (14). Hemoglobin A1c percentage was estimated from whole blood sample, and the estimation of the other analytes was done on serum samples. Hemoglobin A1c percentage was measured by DCA 2000+ Analyzer (Bayer, Inc., Tarrytown, NY, USA) based on Diabetes Control and Complications Trial standards (15). Serum 25-hydroxyvitamin D [25(OH)D] concentration was analyzed using 25-hydroxy chemiluminescent immunoassay (DiaSorin Liaison; Stillwater, Minnesota). Serum lipids were measured at the University of Massachusetts Medical School Clinical Laboratory using the Beckman Coulter AU system which meets the National Cholesterol Education Program’s criteria for accuracy (16). When triglycerides were ≥400 mg/dL, LDL-C was either measured by the beta quantification procedure or calculated by the Friedwald equation (17). Diabetes-associated autoantibodies were measured by Quest Diagnostics, Chantilly, VA, USA. IA-2A and IAA assays were performed using radio-binding assay, while GAD-65 assay was performed using enzyme-linked immunosorbent assay.

### Statistical Analyses

#### Power Calculation

To obtain a power of 80%, at an alpha of 0.05, with a standard deviation of 1.5 using a paired sample analysis, we needed 73 subjects to detect a change in A1c of 0.5%. This sample size was then increased to 110 subjects, further boosting the power of the study to 93% to detect any significant change in A1c, if that change were present.

We first determined the normality of the distribution of each continuous variable using both visual inspection of the histograms of each distribution as well as the Shapiro-Wilk test for normality. If the Shapiro-Wilk test yielded a p-value greater than 0.05, normality of the variable’s distribution (the null hypothesis of the test) was accepted. Some variables exhibited slight deviations from normality, however non-parametric tests such as Kruskal Wallis test confirmed parametric results in these instances and parametric results were reported.

Means and standard deviations (SD) were calculated for the continuous descriptive summary statistics and biochemical parameters. Two-sided Student’s t - test was used to compare the two groups, male and female (**Table 1**); and the two time segments: pre- and post-pandemic lockdown periods. Comparisons of binary variables between the two groups were performed using Pearson’s chi-squared test. General linear models (GLM) were used to analyze the effect of pre-pandemic parameters and other confounders on the post-pandemic endpoint data. GLM was used to determine the relationships between baseline A1c and CGM metrics and other covariates on post-pandemic A1c and CGM metrics. All statistical analyses were performed by using SAS 9.4 Copyright (c) 2016 by SAS Institute Inc., Cary, NC, USA.

**Table 1:** Baseline characteristics of the subjects.

Parameters	All (n=110)	Female (n=53)	Male (n=57)	p* value
Age (year)	14.77 ± 3.9	14.09 ± 3.8	15.41 ± 4.0	0.07
Height z score	0.11 ± 1.1	0.12 ± 1.02	0.10 ± 1.1	0.90
Weight z score	0.86 ± 2.0	0.62 ± 0.9	1.1 ± 2.7	0.22
BMI z score	0.68 ± 1.0	0.72 ± 0.9	0.64 ± 1.2	0.68
Systolic blood pressure (mm Hg)	113.3 ± 10.0	111.65 ± 9.1	115.0 ± 10.7	0.20
Diastolic blood pressure (mm Hg)	72.2 ± 7.0	73.0 ± 6.1	71.4 ± 7.3	0.36
Ethnicity (white, %)	88 (80%)	43 (48.9%)	45 (51.1%)	0.81
Duration of type 1 diabetes (yr)	6.31 ± 4.3	6.4 ± 4.0	6.3 ± 4.6	0.90
Total daily dose of insulin (units/kg/day)	0.93 ± 0.34	0.89 ± 0.29	0.96 ± 0.39	0.34
Hemoglobin A1c (%)	8.86 ± 1.6	8.87 ± 1.7	8.85 ± 1.6	0.98
25-hydroxyvitamin D (ng/mL)	27.83 ± 11.9	28.76 ± 13.3	27.04 ± 10.7	0.65
Total cholesterol (mg/dL)	169.77 ± 29.0	175.19 ± 27.9	164.82 ± 29.8	0.24
LDL cholesterol (mg/dL)	93.70 ± 22.3	96.80 ± 22.3	91.0 ± 22.5	0.39
HDL cholesterol (mg/dL)	56.41 ± 11.3	58.95 ± 12.3	54.09 ± 10.0	0.15
Non-HDL cholesterol (mg/dL)	113.49 ± 26.2	119.00 ± 23.4	108.80 ± 28.2	0.24
VLDL cholesterol (mg/dL)	18.54 ± 7.4	18.96 ± 6.0	18.18 ± 8.5	0.75
Triglycerides (mg/dL)	96.83 ± 44.9	94.35 ± 29.5	99.00 ± 55.6	0.72
TC/HDL ratio	3.03 ± 0.6	2.97 ± 0.6	3.09 ± 0.6	0.56

p* signifies the comparison of the male and female subjects only.

## Results

### Anthropometry and Other Parameters

There were 53 (48.2%) female subjects and 57 (51.8%) male subjects. No significant differences were observed in the baseline parameters between the male and female cohorts (**Table 1**). A review of patients’ medical records showed that no subject tested positive for either an infection with SARS-CoV-2 or an actual COVID-19 disease by any of the available diagnostic techniques including SARS-CoV-2 RNA RT PCR testing.

### Pattern of the Use of Insulin Pump and Glucose Monitoring Devices

Continuous glucose monitoring (CGM) was used by 65 (59.1%) subjects while 33 (30%) were not on a CGM device. Twelve (10.9%) subjects discontinued CGM use during this period, and 27 (24.5%) subjects did not share their CGM data. Therefore, out of the 65 subjects, 38 shared CGM data, and 27 did not share.

Thirty-five (31.8%) subjects wore both CGM and an insulin pump, 11 (10%) used insulin pump only, 31 (28.2%) used CGM only, and 33(30%) used neither CGM nor insulin pump. Subjects who discontinued CGM use during the pandemic, and those who stopped sharing CGM data during the pandemic were excluded from the comparisons between CGM users and non-CGM users.

### Assessment of the Differences in the Change in Point-Of-Care A1c and CGM Metrics Between the Pre- and Post-Pandemic Lockdown Periods

No significant differences were observed in the change in A1c between the pre- and post-lockdown periods, 0.18 ± 1.2%, (95% CI -0.05 to 0.41), p=0.13. Total daily dose of insulin increased in all subjects during the pandemic, 0.04 ± 0.2 units/kg/day, p=0.0093. **Table 2** shows no significant differences in A1c values between the pre- and post-lockdown periods when subjects were stratified by sex, ethnicity, pubertal status, insulin pump usage, and the duration of T1D.

**Table 2:** Differences in the change in point-of-care A1c between the pre- and post-pandemic lockdown periods for all subjects.

Parameters	Differences in the change in pre- and post-lockdown A1c levels (%)	p value
All subjects	0.18 ± 1.2	0.1
Sex		
•Female	0.19 ± 1.2	0.9
•Male	0.16 ± 1.2	
Insulin pump usage		
•Insulin pump users	0.25 ± 1.0	0.5
•Non-insulin pump users	-0.12 ± 1.4	
Pubertal Status		
•Prepubertal	-0.11 ± 0.3	0.6
•Pubertal	0.18 ± 1.3	
Duration of T1D (year)		
•<2	0.2 ± 1.0	0.2
•≥2	-0.22 ± 1.2	
Ethnicity		
•Non-white	0.08 ± 1.6	0.8
•White	0.20 ± 1.1	0.1

### A Sub-Analysis of CGM Usage and Outcome During the Pandemic Lockdown

Subjects who used continuous glucose monitors (CGM) had significant reductions in point of care HbA1c (8.24 ± 1.1% *vs* 7.92 ± 1.1%, p=0.0012) between the pre- and post-pandemic lockdown periods (**Table 3**). The CGM users also showed significant reduction in the estimated A1c, p=(0.0076), mean glucose concentration (p=0.022), and a significant increase in sensor usage (p=0.012), while maintaining no significant change in total daily dose of insulin (TDDI), 0.03 ± 1.3 units/kg/day, p=0.48 (**Table 3**). A sub-analysis of the changes in CGM metrics by covariates showed a significant reduction in A1c in the female subjects, and a near statistically significant reduction in white subjects (**Table 4**).

**Table 3:** Comparison of the differences in CGM metrics between the pre- and post-pandemic lockdown periods among the CGM users only (n=38).

Parameters	Pre-pandemic lockdown period	Post-pandemic lockdown period	p value
Time in range (70-180 mg/dL) (%)	38.29 ± 14.0	42.08 ± 15.3	0.10
Time <70 mg/dL (%)	1.61 ± 1.5	2.02 ± 1.7	0.13
Time <54 mg/dL (%)	0.92 ± 0.4	0.95 ± 0.3	1.0
Time >180 mg/dL (%)	32.76 ± 6.5	30.86 ± 7.6	0.17
Time >250 mg/dL (%)	27.05 ± 14.2	24.37± 14.4	0.09
Coefficient of variation (%)	73.10 ± 12.3	73.23 ± 15.0	0.48
Sensor usage (%)	79.79 ± 23.6	88.68 ± 15.4	**0.014**
Mean glucose (mg/dL)	204.05 ± 31.2	195.95 ± 33.0	**0.0497**
CGM Estimated HbA1c (%)	8.06 ± 0.8	7.92 ± 0.7	**0.018**
Point of Care HbA1c (%)	8.24 ± 1.1	7.90 ± 1.1	**0.0012**
TDDI (unit/kg/day)	0.88 ± 0.3	0.94 ± 0.3	0.48

Pre-pandemic period, Nov 2019 – Feb 2020; post-pandemic lockdown period, July – Nov 2020; HbA1c, hemoglobin A1c; TDDI, total daily dose of insulin; CGM, continuous glucose monitor.

Bolded p values are statistically significant.

**Table 4:** Change in the CGM-estimated HbA1c between the pre- and post-pandemic periods among the CGM users only (n=38).

Parameters	Pre-pandemicCGM estimated A1c	Post-pandemicCGM estimated A1c	p value
Sex			
•Male	8.03 ± 0.8	7.96 ± 0.7	0.60
•Female	8.07 ± 0.7	7.89 ± 0.7	**0.035**
Pubertal status			
•Pre-pubertal	8.11 ± 0.8	7.94 ± 0.7	0.63
•Pubertal	7.82 ± 0.7	7.83 ± 0.8	0.15
Ethnicity			
•White	8.06 ± 0.8	7.89 ± 0.8	0.055
•Non-white	8.00 ± 0.4	8.08 ± 0.6	0.46
Mode of insulin delivery			
•Insulin pump users	7.83 ± 0.6	7.63 ± 0.6	0.48
•Non-pump users	8.54 ± 0.9	8.42 ± 0.7	0.74
Duration of T1D			
•< 2 y	7.50 ± 0.6	7.02 ± 0.5	0.08
•≥ 2 y	8.16 ± 0.7	8.06 ± 0.6	0.78

Pre-pandemic period, Nov 2019 – Feb 2020; post-pandemic lockdown period, July – Nov 2020.

Bolded p values are statistically significant.

The non-CGM cohort, on the other hand, had no significant change in HbA1c between the pre- and post-pandemic periods, 9.48 ± 1.9% versus 9.43 ± 1.6%, p=0.86, even though the non-CGM users had a significant increase in total daily dose of insulin (p<0.0001).

### General Linear Models for Changes in A1c Between the Pre- and Post-Pandemic Lockdown Periods

Using the change in A1c values as the dependent variable, and adjusting the results further for age, sex, BMI z-score, duration of type 1 diabetes, and pump usage, the general linear model (GLM) showed that pre-pandemic A1c was a significant predictor of the value of the change in A1c, p<0.0001. Among the non-glycemic factors: age, sex, BMI, duration of type 1 diabetes, CGM use, and insulin pump usage, only CGM use was a significant predictor of reduction in A1c, p=0.019. The parameters associated with significant reduction in A1c among the CGM users included increased time in range of glycemia, reduction in severe hyperglycemia, increased sensor usage, and reduction in mean glucose concentration (**Table 5**). Serum concentrations of 25-hydroxyvitamin D and lipid parameters were similar between the pre- and post-lockdown periods and were not predictors of change in HbA1c.

**Table 5:** The principal predictors of improved glycemic control in the CGM users during the pandemic lockdown and the resulting change in A1c.

Parameters	Difference between pre- and post-pandemic values	p value
Time in range (%)	-4.6 ± 10.3	**0.035**
Time <70 mg/dL (%)	-0.4 ± 1.4	0.22
Time <54 mg/dL (%)	0.03 ± .05	1.0
Time >180 mg/dL (%)	1.4 ± 6.5	0.32
Time >250 mg/dL (%)	4.0 ± 8.4	**0.02**
Coefficient of variation (%)	1.4 ± 7.3	0.18
Sensor usage (%)	-4.8 ± 11.3	0.059
Mean glucose (mg/dL)	10.7 ± 20.8	**0.014**
TDDI (unit/kg/day)	-0.03 ± 0.1	0.26
TDDI (units/day)	-5.1 ± 6.7	**<0.0001**
CGM-estimated HbA1c (%)	-0.3 ± 0.5	**0.018**
Point of care HbA1c (%)	-0.4 ± 0.5	**<0.0001**

TDDI, total daily dose of insulin; CGM, continuous glucose monitors; significant p values are bolded.

## Discussion

This study reports no significant change in glycemic control during the pandemic lockdown of 2020 in US children. This finding contrasts with the widely expected deterioration in glycemic control during the lockdown. The lack of deterioration in glycemic control in children and adolescents with T1D during the pandemic most likely stemmed from increased parental vigilance, improved adoption of telemedicine for diabetes care, and the role of CGM. The impact of CGM was captured in this study as the CGM users had significant reductions in point-of-care HbA1c, the CGM-estimated A1c, mean glucose concentration, and a significant increase in sensor usage with no change in total daily dose of insulin (TDDI). This suggests that the CGM users precisely targeted and reduced prevalent hyperglycemia without the need to increase their TDDI, which is an indirect evidence for improved compliance with glycemic control.

This study in US children and adolescents also compared the glycemic endpoints in both CGM users and non-CGM users during the pandemic lockdown of 2020. This study is one of the longest reports in this population on the investigation of the effect of the pandemic lockdown on glycemic control in pediatric T1D using A1c as the primary endpoint (9, 18), with supporting data from CGM metrics. This study also provided information on the significant predictors of decreases in A1c during the lockdown.

Our overall finding of no significant change in A1c agrees with earlier reports of no change in glycemic control during the pandemic lockdown (2–4), though our findings in the CGM users agree with studies that reported an improvement in glycemic control (5–8, 19); but disagree with the study that reported worsened glycemic control (9). Possible explanations for these findings include the fact that the worsened glycemic control reported by Verma et al. (9) was due to poor access to insulin during the lockdown in India. Secondly, most of the studies that reported improved glycemic control during the lockdown relied only on short-term indicators of glucose excursions, i.e., CGM metrics, and not A1c. This is an important distinction as previous reports on the effect of the pandemic lockdown on glycemic control in children had focused on either the short-term or medium- to long-term indicators of glycemic control in this population (2, 3, 9, 18, 20). In contrast, this study combined both the short-term and medium- to long-term indicators to examine the effect of the pandemic lockdown on glycemic control in the US.

Though we did not have adequate power to detect significant differences in our sub-groups analysis, some of the reasons for the decrease in A1c in the CGM users could be adduced from the results of the analysis of the CGM metrics. Time in range of glycemia increased from 38% to 42%; time above 250 mg/dL decreased from 27% to 24%, while sensor usage increased from 79% to 88%. Mean glucose level dropped from 204 mg/dL to 195 mg/dL. Female subjects showed a significant decrease in estimated A1c. These positive trends in CGM metrics appear to be associated with significant reductions in both the CGM-estimated A1c and point-of-care A1c. It is possible that wearing CGMs enabled the users to precisely target hyperglycemic episodes with better accuracy than the non-CGM users. These findings in our CGM cohort are similar to our recent report that CGM improves physiologic hyperglycemia of puberty in children and adolescents with T1D (21), as well as a recent report by the T1D Exchange on the impact of CGM on glycemic control (22).

There are other contributing factors to either poor or improved glycemic control that our study did not examine. Some of these factors which could contribute to an optimal glycemic control include an absence of school-related stress in children and adolescents as well as parent; and patients’ apprehension about the unclear outcomes if admitted to the hospital during the lockdown (1). Other factors may include the practice of eating every meal at home with regular timing and close attention to carbohydrate counting under the close supervision of parents or guardians who are also at home with the patient; the practice of engaging in physical activities at home; and the absence of the confounding effect of school and after-school activities (23). Bonora et al. (6) reported that the slowdown of routine activities during the lockdown had a favorable effect on glycemic control in adults. Telemedicine, as a modality of healthcare delivery, may also have played a critical role in preventing adverse outcomes in patients with type 1 diabetes during the pandemic lockdown (24). Telemedicine, which provides a crucial bridge between the patients and their caregivers, is a veritable modality for delivering effective diabetes care (25).

This study has some strengths and limitations that should be taken into consideration in the interpretation of the results. The first limitation was the study’s retrospective design which precluded any allusion to causality between the parameters studied. Though our study was powered to detect a difference in the entire study population, it was not adequately powered to detect differences in the CGM cohort as some of the subjects using CGM were either not sharing glycemic data with the healthcare team or discontinued the use of their device during the pandemic lockdown. We did not have A1c values during the pandemic lockdown because patients were not coming to the clinic. This would have provided us with the actual A1c data during this critical period. However, the A1c values obtained 2-4 months after the pandemic lockdown reflected the glycemic trends in the preceding 120 days which coincided with the period of the pandemic lockdown. Because the subjects were not randomly selected for inclusion in the study, it is possible that our cohort represents subjects with the best glycemic control. Subjects’ home environment, eating habits, and exercise patterns were not documented during the lockdown, so it was not possible to investigate the effect of these factors on our results. The strengths of this study included an adequate statistical power of 93% to detect any significant differences in A1c. Further strengths of the study included the use of a representative sample of children and adolescents with T1D with respect to sex, ethnicity, age, duration of type 1 diabetes, and BMI status. The longitudinal nature of the data allowed each patient to serve as their own control. The adjustment of the results for core confounders enhanced the validity of the results. The inclusion of CGM metrics enabled for a granular characterization of the dichotomy in glycemic outcomes between the CGM users and non-CGM users. This study advances the field by clarifying the nature of glycemic excursions in pediatric patients with T1D during the pandemic lockdown of 2020 in the US.

In conclusion, this study found no overall change in glycemic control during the pandemic lockdown of 2020 in US children and adolescents with T1D. There was, however, a significant reduction in A1c in the CGM users though the study was not adequately powered to detect differences in subgroup analysis. These results from a major teaching hospital in the US, provide more scientific data on the impact of the pandemic lockdown on glycemic control, and could help in the planning of approaches to glycemic control in future lockdown scenarios.

## Data Availability Statement

The original contributions presented in the study are included in the article/supplementary material. Further inquiries can be directed to the corresponding author.

## Ethics Statement

The studies involving human participants were reviewed and approved by University of Massachusetts Institutional Review Board. Written informed consent from the participants’ legal guardian/next of kin was not required to participate in this study in accordance with the national legislation and the institutional requirements.

## Author Contributions

All the authors have accepted responsibility for the entire content of this submitted manuscript and approved submission. BN conceived the project, researched data, and wrote the manuscript; SP researched data; LA-H researched data; GJ researched data. EZ-M and BB performed statistical analysis. All authors contributed to the article and approved the submitted version. BN is the guarantor of this manuscript and takes full responsibility for the work as a whole.

## Funding

This study was supported in part by an investigator-initiated research grant, Grant ID: 1 R21 DK113353-01, to BN from NIDDK, NIH.

## Conflict of Interest

The authors declare that the research was conducted in the absence of any commercial or financial relationships that could be construed as a potential conflict of interest.

## Publisher’s Note

All claims expressed in this article are solely those of the authors and do not necessarily represent those of their affiliated organizations, or those of the publisher, the editors and the reviewers. Any product that may be evaluated in this article, or claim that may be made by its manufacturer, is not guaranteed or endorsed by the publisher.
